# Isopropyl 2-[5-(4-hydroxy­phen­yl)-3-methyl­sulfanyl-1-benzofuran-2-yl]acetate

**DOI:** 10.1107/S1600536809033492

**Published:** 2009-08-29

**Authors:** Hong Dae Choi, Pil Ja Seo, Byeng Wha Son, Uk Lee

**Affiliations:** a Department of Chemistry, Dongeui University, San 24 Kaya-dong Busanjin-gu, Busan 614-714, Republic of Korea; b Department of Chemistry, Pukyong National University, 599-1 Daeyeon 3-dong, Nam-gu, Busan 608-737, Republic of Korea

## Abstract

In the title compound, C_20_H_20_O_4_S, the 4-hydroxy­phenyl ring is rotated out of the plane of the benzofuran unit by 32.87 (8)°. The S—C_meth­yl_ bond is almost perpendicular to the plane of the benzofuran fragment [77.8 (1)°] and is slightly tilted towards it. The crystal structure is stabilized by inter­molecular O—H⋯O and C—H⋯O hydrogen bonds.

## Related literature

For the crystal structure of a similar alkyl 2-[5-(4-hydroxy­phen­yl)-3-methyl­sulfanyl-1-benzofuran-2-yl]acetate derivative, see: Choi *et al.* (2006 ▶). For the pharmacological activity of benzofuran compounds, see: Howlett *et al.* (1999 ▶); Twyman & Allsop (1999 ▶). For natural products containing the benzofuran unit, see: Akgul & Anil (2003 ▶); von Reuss & König (2004 ▶).
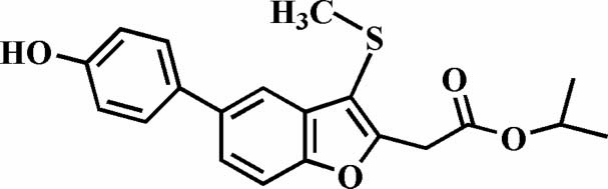


## Experimental

### 

#### Crystal data

C_20_H_20_O_4_S
*M* *_r_* = 356.42
Monoclinic, 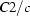

*a* = 31.375 (3) Å
*b* = 8.0055 (7) Å
*c* = 15.274 (1) Å
β = 107.727 (1)°
*V* = 3654.2 (5) Å^3^
*Z* = 8
Mo *K*α radiation
μ = 0.20 mm^−1^
*T* = 173 K
0.45 × 0.40 × 0.10 mm

#### Data collection

Bruker SMART CCD diffractometer
Absorption correction: multi-scan (*SADABS*; Sheldrick, 2000 ▶) *T*_min_ = 0.916, *T*_max_ = 0.981
15589 measured reflections
4158 independent reflections
2474 reflections with *I* > 2σ(*I*)
*R*_int_ = 0.051

#### Refinement

*R*[*F* ^2^ > 2σ(*F* ^2^)] = 0.042
*wR*(*F* ^2^) = 0.111
*S* = 1.06
4158 reflections
233 parameters
H atoms treated by a mixture of independent and constrained refinement
Δρ_max_ = 0.17 e Å^−3^
Δρ_min_ = −0.25 e Å^−3^

### 

Data collection: *SMART* (Bruker, 2001 ▶); cell refinement: *SAINT* (Bruker, 2001 ▶); data reduction: *SAINT*; program(s) used to solve structure: *SHELXS97* (Sheldrick, 2008 ▶); program(s) used to refine structure: *SHELXL97* (Sheldrick, 2008 ▶); molecular graphics: *ORTEP-3* (Farrugia, 1997 ▶) and *DIAMOND* (Brandenburg, 1998 ▶); software used to prepare material for publication: *SHELXL97*.

## Supplementary Material

Crystal structure: contains datablocks global, I. DOI: 10.1107/S1600536809033492/bt5043sup1.cif

Structure factors: contains datablocks I. DOI: 10.1107/S1600536809033492/bt5043Isup2.hkl

Additional supplementary materials:  crystallographic information; 3D view; checkCIF report

## Figures and Tables

**Table 1:** Hydrogen-bond geometry (Å, °)

*D*—H⋯*A*	*D*—H	H⋯*A*	*D*⋯*A*	*D*—H⋯*A*
O4—H4⋯O3^i^	0.81 (3)	2.02 (3)	2.829 (3)	171 (3)
C13—H13*B*⋯O4^ii^	0.96	2.57	3.325 (3)	135

Symmetry codes: (i) 
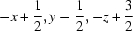
; (ii) 
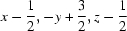
.

**Fig. 1.**
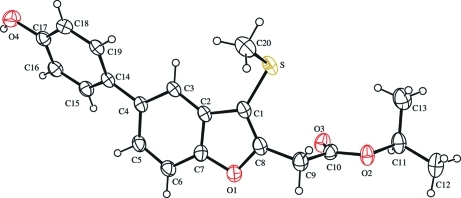
The molecular structure of the title compound with the atom numbering scheme. Displacement ellipsoids are drawn at the 50% probability level. H atoms are presented as a small cycles of arbitrary radius.

**Fig. 2.**
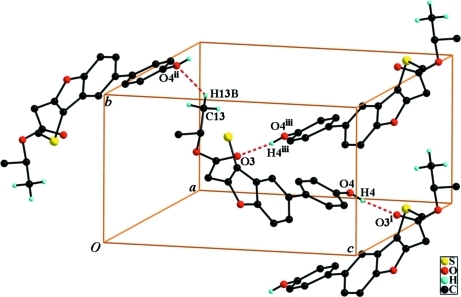
O–H···O and C–H···O interactions (dotted lines) in the title compound. [Symmetry codes: (i) - x + 1/2, y - 1/2, - z + 3/2; (ii) x - 1/2, - y + 3/2, z - 1/2 (iii) -x + 1/2, y + 1/2, - z+ 3/2.]

## supplementary crystallographic information

### Comment

Hetero aromatic compounds containing the benzofuran skeleton have attracted particular interest in the view of their pharmacological properties (Howlett *et al.*, 1999; Twyman & Allsop, 1999), and these compounds are occurring in natural products (Akgul & Anil, 2003; von Reuss & König, 2004). As a part of our ongoing studies of the effect of side chain substituents on the solid state structures of alkyl 2-[5-(4-hydroxyphenyl)-3-methylsulfanyl-1-benzofuran-2-yl]acetate analogues (Choi *et al.*, 2006), the crystal structure of the title compound has been determined (Fig. 1).

The benzofuran unit is essentially planar, with a mean deviation of 0.007 (2) Å from the least-squares plane defined by the nine constituent atoms. The 4-hydroxyphenyl ring is rotated out of the benzofuran plane, with a dihedral angle of 32.87 (8)°. The crystal packing (Fig. 2) is stabilized by intermolecular O–H···O and C–H···O hydrogen bonds; the first between the H atom of the hydroxy group and the oxygen of the C═O unit, with a O4–H4···O3^i^, the second between the methyl H atom of the methylsulfinyl substituent and the H atom of the hydroxy group, with a C13–H13B···O4^ii^, respectively (Table 1).

### Experimental

2-[5-(4-Hydroxyphenyl)-3-methylsulfanyl-1-benzofuran-2-yl]acetic acid (377 mg, 1.2 mmol) was added to a solution of concentrated sulfuric acid (3 drops) in isopropanol (15 ml), and the mixture was refluxed for 6h, then cooled. The solvent was evaporated and the residue was poured into water. The mixture was extracted with dichloromethane, dried over magnesium sulfate, filtered and concentrated under vacuum. The residue was purified by column chromatography (benzene-acetone, 9:1 v/v) to afford the title compound as a colorless solid [yield 91%, m.p. 410-411 K; *R*_f_ = 0.51 (benzene-acetone, 9:1 v/v)]. Single crystals suitable for X–ray diffraction were prepared by evaporation of a solution of the title compound in benzene at room temperature.

### Refinement

The hydroxyl H atom was found in a difference Fourier map and refined freely. The other H atoms were positioned geometrically and refined using a riding model, with C–H = 0.93 Å for the aryl, 0.97 Å for the methine and methylene, and 0.96 Å for the methyl H atoms. *U*_iso_(H) = 1.2*U*_eq_(C) for the aryl, methine  and methylene H atoms, and 1.5*U*_eq_(C) for methyl H atoms.

### Crystal data

**Table:**

C_20_H_20_O_4_S	*F*(000) = 1504
*M**_r_* = 356.42	*D*_x_ = 1.296 Mg m^−^^3^
Monoclinic, *C*2/*c*	Mo *K*α radiation, λ = 0.71073 Å
Hall symbol: -C 2yc	Cell parameters from 4594 reflections
*a* = 31.375 (3) Å	θ = 2.6–27.3°
*b* = 8.0055 (7) Å	µ = 0.20 mm^−^^1^
*c* = 15.274 (1) Å	*T* = 173 K
β = 107.727 (1)°	Block, colorless
*V* = 3654.2 (5) Å^3^	0.45 × 0.40 × 0.10 mm
*Z* = 8	

### Data collection

**Table:**

Bruker SMART CCD diffractometer	4158 independent reflections
Radiation source: fine-focus sealed tube	2474 reflections with *I* > 2σ(*I*)
graphite	*R*_int_ = 0.051
Detector resolution: 10.0 pixels mm^-1^	θ_max_ = 27.5°, θ_min_ = 1.4°
φ and ω scans	*h* = −39→40
Absorption correction: multi-scan (*SADABS*; Sheldrick, 2000)	*k* = −10→10
*T*_min_ = 0.916, *T*_max_ = 0.981	*l* = −19→19
15589 measured reflections	

### Refinement

**Table:**

Refinement on *F*^2^	Primary atom site location: structure-invariant direct methods
Least-squares matrix: full	Secondary atom site location: difference Fourier map
*R*[*F*^2^ > 2σ(*F*^2^)] = 0.042	Hydrogen site location: difference Fourier map
*wR*(*F*^2^) = 0.111	H atoms treated by a mixture of independent and constrained refinement
*S* = 1.06	*w* = 1/[σ^2^(*F*_o_^2^) + (0.024*P*)^2^ + 3.8686*P*] where *P* = (*F*_o_^2^ + 2*F*_c_^2^)/3
4158 reflections	(Δ/σ)_max_ < 0.001
233 parameters	Δρ_max_ = 0.17 e Å^−^^3^
0 restraints	Δρ_min_ = −0.24 e Å^−^^3^

### Special details

**Table:**

Geometry. All esds (except the esd in the dihedral angle between two l.s. planes) are estimated using the full covariance matrix. The cell esds are taken into account individually in the estimation of esds in distances, angles and torsion angles; correlations between esds in cell parameters are only used when they are defined by crystal symmetry. An approximate (isotropic) treatment of cell esds is used for estimating esds involving l.s. planes.
Refinement. Refinement of F^2^ against ALL reflections. The weighted R-factor wR and goodness of fit S are based on F^2^, conventional R-factors R are based on F, with F set to zero for negative F^2^. The threshold expression of F^2^ > 2sigma(F^2^) is used only for calculating R-factors(gt) etc. and is not relevant to the choice of reflections for refinement. R-factors based on F^2^ are statistically about twice as large as those based on F, and R- factors based on ALL data will be even larger.

### Fractional atomic coordinates and isotropic or equivalent isotropic displacement parameters (Å^2^)

**Table:**

	*x*	*y*	*z*	*U*_iso_*/*U*_eq_	
S	0.15080 (2)	0.69811 (7)	0.43431 (4)	0.0571 (2)	
O1	0.11004 (5)	0.2623 (2)	0.49946 (11)	0.0540 (4)	
O2	0.00249 (5)	0.6432 (2)	0.36507 (10)	0.0547 (4)	
O3	0.04570 (5)	0.6124 (2)	0.51076 (10)	0.0588 (5)	
O4	0.43182 (6)	0.2543 (2)	0.81139 (14)	0.0639 (5)	
H4	0.4366 (11)	0.204 (4)	0.860 (2)	0.097 (12)*	
C1	0.14460 (8)	0.4954 (3)	0.47235 (15)	0.0471 (6)	
C2	0.17779 (8)	0.3928 (3)	0.53678 (14)	0.0430 (5)	
C3	0.22327 (7)	0.4045 (3)	0.58158 (14)	0.0427 (5)	
H3	0.2393	0.4973	0.5728	0.051*	
C4	0.24484 (7)	0.2758 (3)	0.64005 (14)	0.0421 (5)	
C5	0.21960 (8)	0.1362 (3)	0.65124 (16)	0.0501 (6)	
H5	0.2341	0.0503	0.6899	0.060*	
C6	0.17442 (8)	0.1219 (3)	0.60733 (16)	0.0531 (6)	
H6	0.1582	0.0293	0.6156	0.064*	
C7	0.15455 (8)	0.2522 (3)	0.55045 (16)	0.0475 (6)	
C8	0.10569 (8)	0.4117 (3)	0.45225 (16)	0.0517 (6)	
C9	0.06007 (8)	0.4503 (3)	0.39027 (16)	0.0571 (6)	
H9A	0.0427	0.3480	0.3775	0.069*	
H9B	0.0625	0.4924	0.3324	0.069*	
C10	0.03578 (7)	0.5767 (3)	0.43027 (15)	0.0459 (5)	
C11	−0.02201 (8)	0.7816 (3)	0.39016 (17)	0.0596 (7)	
H11	−0.0251	0.7615	0.4512	0.072*	
C12	−0.06727 (9)	0.7810 (4)	0.31955 (19)	0.0779 (9)	
H12A	−0.0640	0.7979	0.2597	0.117*	
H12B	−0.0852	0.8692	0.3327	0.117*	
H12C	−0.0816	0.6756	0.3211	0.117*	
C13	0.00498 (10)	0.9398 (4)	0.3919 (2)	0.0810 (9)	
H13A	0.0336	0.9296	0.4379	0.121*	
H13B	−0.0108	1.0337	0.4060	0.121*	
H13C	0.0092	0.9561	0.3328	0.121*	
C14	0.29366 (7)	0.2806 (2)	0.68845 (14)	0.0412 (5)	
C15	0.31181 (8)	0.2046 (3)	0.77431 (15)	0.0482 (6)	
H15	0.2927	0.1583	0.8037	0.058*	
C16	0.35724 (8)	0.1966 (3)	0.81618 (15)	0.0489 (6)	
H16	0.3684	0.1444	0.8730	0.059*	
C17	0.38628 (8)	0.2652 (3)	0.77459 (15)	0.0462 (5)	
C18	0.36918 (8)	0.3480 (3)	0.69133 (15)	0.0472 (5)	
H18	0.3885	0.3992	0.6640	0.057*	
C19	0.32372 (7)	0.3543 (2)	0.64927 (14)	0.0429 (5)	
H19	0.3128	0.4091	0.5932	0.051*	
C20	0.18438 (10)	0.6628 (3)	0.3595 (2)	0.0753 (8)	
H20A	0.2132	0.6210	0.3947	0.113*	
H20B	0.1880	0.7659	0.3306	0.113*	
H20C	0.1699	0.5825	0.3134	0.113*	

### Atomic displacement parameters (Å^2^)

**Table:**

	*U*^11^	*U*^22^	*U*^33^	*U*^12^	*U*^13^	*U*^23^
S	0.0746 (5)	0.0372 (3)	0.0542 (4)	0.0154 (3)	0.0118 (3)	0.0026 (3)
O1	0.0501 (10)	0.0527 (10)	0.0582 (10)	0.0071 (8)	0.0151 (8)	0.0052 (8)
O2	0.0574 (10)	0.0634 (10)	0.0371 (8)	0.0202 (8)	0.0051 (7)	−0.0031 (7)
O3	0.0536 (10)	0.0758 (12)	0.0392 (9)	0.0075 (9)	0.0023 (8)	−0.0064 (8)
O4	0.0525 (11)	0.0710 (12)	0.0578 (12)	−0.0048 (9)	0.0013 (9)	0.0086 (10)
C1	0.0551 (15)	0.0395 (12)	0.0441 (13)	0.0103 (11)	0.0113 (11)	−0.0003 (10)
C2	0.0521 (14)	0.0363 (11)	0.0416 (12)	0.0091 (10)	0.0157 (10)	0.0003 (9)
C3	0.0533 (14)	0.0339 (11)	0.0420 (12)	0.0062 (10)	0.0162 (11)	0.0003 (9)
C4	0.0509 (13)	0.0382 (11)	0.0396 (12)	0.0093 (10)	0.0176 (10)	0.0027 (9)
C5	0.0528 (15)	0.0460 (13)	0.0554 (14)	0.0114 (11)	0.0223 (12)	0.0146 (11)
C6	0.0553 (16)	0.0467 (13)	0.0628 (15)	0.0053 (11)	0.0262 (13)	0.0137 (12)
C7	0.0440 (14)	0.0493 (13)	0.0505 (13)	0.0080 (10)	0.0162 (11)	0.0023 (11)
C8	0.0576 (16)	0.0459 (13)	0.0490 (14)	0.0128 (12)	0.0124 (12)	−0.0014 (11)
C9	0.0569 (15)	0.0560 (15)	0.0518 (14)	0.0118 (12)	0.0066 (12)	−0.0075 (12)
C10	0.0420 (13)	0.0497 (13)	0.0405 (13)	0.0015 (10)	0.0043 (10)	−0.0011 (10)
C11	0.0624 (16)	0.0686 (17)	0.0468 (14)	0.0251 (14)	0.0150 (12)	−0.0004 (12)
C12	0.0672 (19)	0.096 (2)	0.0622 (17)	0.0311 (17)	0.0075 (14)	0.0046 (16)
C13	0.092 (2)	0.0672 (19)	0.078 (2)	0.0169 (17)	0.0178 (18)	−0.0034 (15)
C14	0.0531 (14)	0.0326 (11)	0.0390 (11)	0.0071 (10)	0.0154 (10)	0.0007 (9)
C15	0.0576 (15)	0.0453 (12)	0.0441 (12)	0.0056 (11)	0.0190 (11)	0.0084 (11)
C16	0.0592 (15)	0.0457 (13)	0.0381 (12)	0.0053 (11)	0.0090 (11)	0.0048 (10)
C17	0.0494 (14)	0.0392 (12)	0.0444 (12)	0.0006 (10)	0.0059 (11)	−0.0040 (10)
C18	0.0560 (15)	0.0417 (12)	0.0447 (13)	−0.0034 (11)	0.0164 (11)	−0.0004 (10)
C19	0.0558 (15)	0.0349 (11)	0.0373 (11)	0.0057 (10)	0.0133 (11)	0.0033 (9)
C20	0.099 (2)	0.0535 (16)	0.0803 (19)	0.0075 (15)	0.0382 (18)	0.0049 (14)

### Geometric parameters (Å, °)

**Table:**

S—C1	1.754 (2)	C9—H9B	0.9700
S—C20	1.796 (3)	C11—C12	1.499 (3)
O1—C7	1.379 (3)	C11—C13	1.519 (4)
O1—C8	1.381 (3)	C11—H11	0.9800
O2—C10	1.316 (3)	C12—H12A	0.9600
O2—C11	1.465 (3)	C12—H12B	0.9600
O3—C10	1.207 (2)	C12—H12C	0.9600
O4—C17	1.370 (3)	C13—H13A	0.9600
O4—H4	0.81 (3)	C13—H13B	0.9600
C1—C8	1.344 (3)	C13—H13C	0.9600
C1—C2	1.450 (3)	C14—C19	1.393 (3)
C2—C3	1.385 (3)	C14—C15	1.400 (3)
C2—C7	1.391 (3)	C15—C16	1.374 (3)
C3—C4	1.396 (3)	C15—H15	0.9300
C3—H3	0.9300	C16—C17	1.374 (3)
C4—C5	1.409 (3)	C16—H16	0.9300
C4—C14	1.485 (3)	C17—C18	1.389 (3)
C5—C6	1.376 (3)	C18—C19	1.375 (3)
C5—H5	0.9300	C18—H18	0.9300
C6—C7	1.379 (3)	C19—H19	0.9300
C6—H6	0.9300	C20—H20A	0.9600
C8—C9	1.489 (3)	C20—H20B	0.9600
C9—C10	1.504 (3)	C20—H20C	0.9600
C9—H9A	0.9700		
			
C1—S—C20	102.15 (11)	O2—C11—H11	109.7
C7—O1—C8	105.56 (18)	C12—C11—H11	109.7
C10—O2—C11	117.84 (17)	C13—C11—H11	109.7
C17—O4—H4	106 (2)	C11—C12—H12A	109.5
C8—C1—C2	106.7 (2)	C11—C12—H12B	109.5
C8—C1—S	124.77 (18)	H12A—C12—H12B	109.5
C2—C1—S	128.29 (18)	C11—C12—H12C	109.5
C3—C2—C7	119.2 (2)	H12A—C12—H12C	109.5
C3—C2—C1	135.7 (2)	H12B—C12—H12C	109.5
C7—C2—C1	105.0 (2)	C11—C13—H13A	109.5
C2—C3—C4	119.5 (2)	C11—C13—H13B	109.5
C2—C3—H3	120.3	H13A—C13—H13B	109.5
C4—C3—H3	120.3	C11—C13—H13C	109.5
C3—C4—C5	118.8 (2)	H13A—C13—H13C	109.5
C3—C4—C14	121.7 (2)	H13B—C13—H13C	109.5
C5—C4—C14	119.53 (19)	C19—C14—C15	116.8 (2)
C6—C5—C4	122.7 (2)	C19—C14—C4	121.87 (19)
C6—C5—H5	118.6	C15—C14—C4	121.2 (2)
C4—C5—H5	118.6	C16—C15—C14	121.6 (2)
C5—C6—C7	116.4 (2)	C16—C15—H15	119.2
C5—C6—H6	121.8	C14—C15—H15	119.2
C7—C6—H6	121.8	C17—C16—C15	120.4 (2)
O1—C7—C6	125.9 (2)	C17—C16—H16	119.8
O1—C7—C2	110.76 (19)	C15—C16—H16	119.8
C6—C7—C2	123.3 (2)	O4—C17—C16	122.7 (2)
C1—C8—O1	112.0 (2)	O4—C17—C18	118.0 (2)
C1—C8—C9	132.3 (2)	C16—C17—C18	119.2 (2)
O1—C8—C9	115.8 (2)	C19—C18—C17	120.1 (2)
C8—C9—C10	112.92 (19)	C19—C18—H18	119.9
C8—C9—H9A	109.0	C17—C18—H18	119.9
C10—C9—H9A	109.0	C18—C19—C14	121.7 (2)
C8—C9—H9B	109.0	C18—C19—H19	119.2
C10—C9—H9B	109.0	C14—C19—H19	119.2
H9A—C9—H9B	107.8	S—C20—H20A	109.5
O3—C10—O2	125.0 (2)	S—C20—H20B	109.5
O3—C10—C9	124.6 (2)	H20A—C20—H20B	109.5
O2—C10—C9	110.39 (19)	S—C20—H20C	109.5
O2—C11—C12	105.5 (2)	H20A—C20—H20C	109.5
O2—C11—C13	107.5 (2)	H20B—C20—H20C	109.5
C12—C11—C13	114.6 (2)		
			
C20—S—C1—C8	−113.8 (2)	C7—O1—C8—C1	−1.1 (2)
C20—S—C1—C2	72.8 (2)	C7—O1—C8—C9	179.05 (19)
C8—C1—C2—C3	178.3 (2)	C1—C8—C9—C10	−77.0 (3)
S—C1—C2—C3	−7.3 (4)	O1—C8—C9—C10	102.7 (2)
C8—C1—C2—C7	−0.6 (2)	C11—O2—C10—O3	5.8 (4)
S—C1—C2—C7	173.76 (17)	C11—O2—C10—C9	−173.8 (2)
C7—C2—C3—C4	−0.5 (3)	C8—C9—C10—O3	−18.4 (4)
C1—C2—C3—C4	−179.3 (2)	C8—C9—C10—O2	161.2 (2)
C2—C3—C4—C5	0.5 (3)	C10—O2—C11—C12	−155.9 (2)
C2—C3—C4—C14	178.91 (19)	C10—O2—C11—C13	81.4 (3)
C3—C4—C5—C6	−0.4 (3)	C3—C4—C14—C19	−33.2 (3)
C14—C4—C5—C6	−178.9 (2)	C5—C4—C14—C19	145.2 (2)
C4—C5—C6—C7	0.3 (3)	C3—C4—C14—C15	149.6 (2)
C8—O1—C7—C6	−178.9 (2)	C5—C4—C14—C15	−32.0 (3)
C8—O1—C7—C2	0.7 (2)	C19—C14—C15—C16	−2.7 (3)
C5—C6—C7—O1	179.3 (2)	C4—C14—C15—C16	174.6 (2)
C5—C6—C7—C2	−0.3 (3)	C14—C15—C16—C17	0.6 (3)
C3—C2—C7—O1	−179.24 (18)	C15—C16—C17—O4	−177.1 (2)
C1—C2—C7—O1	−0.1 (2)	C15—C16—C17—C18	2.3 (3)
C3—C2—C7—C6	0.4 (3)	O4—C17—C18—C19	176.5 (2)
C1—C2—C7—C6	179.5 (2)	C16—C17—C18—C19	−2.9 (3)
C2—C1—C8—O1	1.1 (3)	C17—C18—C19—C14	0.7 (3)
S—C1—C8—O1	−173.51 (15)	C15—C14—C19—C18	2.1 (3)
C2—C1—C8—C9	−179.1 (2)	C4—C14—C19—C18	−175.23 (19)
S—C1—C8—C9	6.3 (4)		

### Hydrogen-bond geometry (Å, °)

**Table:**

*D*—H···*A*	*D*—H	H···*A*	*D*···*A*	*D*—H···*A*
O4—H4···O3^i^	0.81 (3)	2.02 (3)	2.829 (3)	171 (3)
C13—H13B···O4^ii^	0.96	2.57	3.325 (3)	135

Symmetry codes: (i) −*x*+1/2, *y*−1/2, −*z*+3/2; (ii) *x*−1/2, −*y*+3/2, *z*−1/2.
